# Comparative transcriptomic insights into the domestication of *Pleurotus abieticola* for coniferous cultivation

**DOI:** 10.3389/fmolb.2023.1352163

**Published:** 2024-01-10

**Authors:** Lei Sun, Frederick Leo Sossah, Yu Li, Huiying Sun

**Affiliations:** ^1^ International Cooperation Research Center of China for New Germplasm Breeding of Edible Mushrooms, Jilin Agricultural University, Changchun, Jilin, China; ^2^ Jilin Province Key Laboratory of Fungal Phenomics, Jilin Agricultural University, Changchun, Jilin, China; ^3^ Council for Scientific and Industrial Research (CSIR), Oil Palm Research Institute, Coconut Research Programme, Sekondi, Ghana

**Keywords:** *Pleurotus abieticola*, lignocellulose degradation, cultivation optimization, conifer adaptation, biotechnological potential, sustainable agriculture

## Abstract

**Introduction:** *Pleurotus abieticola*, a promising edible fungus in the Pleurotaceae family, especially its ability to utilize coniferous substrate, holds significant potential for commercial cultivation. However, few reports on the adaptation of *P. abieticola* to coniferous substrate from the perspective of omics.

**Methods:** This study explores the biological characteristics, domestication process, and nutritional composition of *P. abieticola*, along with its adaptability to coniferous substrates using transcriptomics. We assessed biological characteristics, optimizing mycelial growth on agar medium with varied carbon and nitrogen sources, temperature, and pH. Additionally, the optimization process extended to fruiting bodies, where impact on the differentiation were evaluated under varying light conditions. Fruiting body nutrient composition was analyzed per the Chinese National Food Safety Standard. Transcriptome sequencing focused on *P. abieticola* mycelial colonized coniferous and broadleaved substrates.

**Results and Discussion:** The optimal conditions for mycelial growth were identified: dextrin (carbon source), diammonium hydrogen phosphate (nitrogen source), 25°C (temperature), and pH 7.0. White light promoted fruiting body growth and differentiation. Larch substrate exhibited superior yield (190 g) and biological efficiency (38.0%) compared to oak (131 g, 26.2%) and spruce (166 g, 33.2%). *P. abieticola* showcased high dietary fiber, protein, and total sugar content, low fat, and sufficient microelements. Transcriptome analysis revealed significant key genes involved in lignocellulose degradation, stress-resistant metabolism, and endocytosis metabolism, underscoring their pivotal for coniferous adaptation. This study offers valuable insights for the commercial development and strain breeding of *P. abieticola*, efficiently leveraging conifer resources. The findings underscore its potential as a valuable source for food, medicinal products, and biotechnological applications.

## 1 Introduction

Lignocellulose biomass is a valuable resource for renewable energy and chemical production (Ashokkumar et al., 2022). It is made up of three main components: cellulose, hemicellulose, and lignin, and it is generated from a variety of sources, including agriculture, forestry, and industrial processes (Wang et al., 2021). Forests and forest plantations are significant sources of lignocellulose waste generated from logging residues, sawmill waste, and other wood-processing activities. Similarly, agricultural residues such as corn stalks, wheat straws, and rice husks are also rich in lignocellulose and can be used as a source of biomass for bioenergy production. In addition to these sources, municipal solid, food, and yard waste can also provide significant amounts of lignocellulose waste.

Traditionally, broad-leaved trees have been the primary substrate for wood-decaying mushroom cultivation, with added auxiliary materials such as wheat bran (Li et al., 2020; Doroški et al., 2022; Hao et al., 2022; Yu et al., 2022). With the concept of “forest protection” gradually deepens into people’s minds, wood logging, especially hardwood, is gradually decreasing, which has caused a substrate shortage for the use of broad-leaved sawdust to cultivate edible mushrooms. Conifers are a major component of forest resources in China, accounting for around 70% of the total arbor resources (Cui and Liu, 2020). However, they contain terpenoids and phenols that can impede the growth and development of mycelia and fruiting bodies of edible mushrooms (Adrian et al., 2022). While conifers can be used for mushroom production after treatment, the complex treatment process and high cost limit their use as a substrate.

The *Pleurotus* genus is the most extensively domesticated among the cultivated macrofungi, with more than ten species successfully domesticated for food, medicine, and industrial purposes (Ihaka and Gentleman, 1996; Hu et al., 2018). The genus is also among the top three in yield worldwide, with more than 7 million tons in China (Zhang et al., 2017; Li et al., 2019). While many fungal species in the *Pleurotus* genus can grow and form fruiting bodies on fungus-treated coniferous wood, such as *P*. *euosmus*, *Pleurotus eryngii*, *Pleurotus ostreatus*, *P*. *populinus*, and *Pleurotus pulmonarius*, the treatment process incurs high costs (Guo et al., 2021). If *Pleurotus* species can directly use coniferous substrate without treatment, it could help reduce production costs and increase income.

*P*. *abieticola*, a fungus belonging to the *Pleurotus* genus, is remarkable for its ability to grow on both broadleaf *Betula* coniferous *Picea* substrates, making it a versatile species for cultivation (Liu et al., 2015). Apart from its adaptability, *P*. *abieticola* also possesses significant nutritional and medicinal properties that enhance its potential value. Its polysaccharides, triterpenoids, and ergosterol have been found to have numerous pharmacological activities, including antioxidant, anti-inflammatory, and immunomodulatory effects (Zheng et al., 2021; Pan et al., 2022). These unique properties make *P*. *abieticola* a promising candidate for use in both functional food ingredients and pharmaceutical applications.

The aim of this study was to investigate the adaptability of *P*. *abieticola* to coniferous trees. To achieve this, the mycelia of *P*. *abieticola* were cultivated on both coniferous and broad-leaved substrates, and transcriptome sequencing was conducted to identify and analyze differentially expressed genes under these different substrate conditions. The study aimed to understand the functional genes and their expression patterns involved in the adaptation to coniferous and broad-leaved tree substrate utilization at the transcription level. The ultimate goal was to provide a theoretical foundation for the rational utilization and development of new varieties of *P*. *abieticola*. In addition, this study characterized the biological characteristics and nutritional composition of *P*. *abieticola* and established cultivation techniques for its domestication and commercialization. Furthermore, transcriptome analysis was conducted to elucidate the mechanisms underlying its ability to utilize conifers as a substrate.

## 2 Material and methods

### 2.1 Strain selection and preparation

The wild strain of *P*. *abieticola* used in this study was obtained from the Herbarium of Mycology of Jilin Agricultural University. The oak sawdust, spruce sawdust, larch sawdust, and wheat bran were obtained from the Mushroom base of Jilin Agricultural University, China. The wild strain of *P*. *abieticola* was isolated and inoculated onto a potato dextrose agar (PDA) solid medium under aseptic conditions. The mycelia were incubated at 25°C for 15 days in the dark. The mycelia were allowed to grow to a length of 7–8 cm for strain expansion.

### 2.2 Optimization of mycelial growth

To optimize the growth conditions of *P*. *abieticola*, glucose, sucrose, soluble starch, and dextrin were used as the carbon source, while peptone, yeast powder, diammonium hydrogen phosphate [(NH_4_)_2_HPO_4_], and ammonium chloride (NH_4_Cl) was used as the nitrogen source. The culture medium was adjusted to different pH levels of 5.0, 6.0, 7.0, 8.0, and 9.0, and incubation temperatures of 15°C, 20°C, 25°C, 30°C, and 35°C were used. The detailed cultivation formula is provided in Supplementary Table S1.

After sterilization at 121°C for 30 min, the different culture mediums were poured into 90 mm Petri dishes and inoculated with the strains after they solidified. The plates were incubated at 25°C in the dark, and the colony morphology (including colony color, colony edge uniformity, mycelia growth vitality, and other characteristics) was recorded every 48 h after inoculation. The colony diameter was measured by the cross method, and the growth rate of mycelium was calculated as

$$Mycelium\;growth\;rate=\frac{Colony\;radius}{Number\;of\;culture\;days}$$

Each treatment was performed three times. The analysis of variance was performed using SPSS software (Rode and Ringel, 2019).

### 2.3 Domestication and cultivation

After filling individual polypropylene bags with 800 g of each substrate with moisture content 60% (Supplementary Table S1), they were autoclaved at 121°C for 2 h and cooled to room temperature before inoculating each bag with 10 mL of *P*. *abieticola* liquid spawn. For each substrate, 18 bags were inoculated and arranged in a completely randomized design. Once the spawn running process was complete, all spawn was transferred to a mushroom room with controlled environmental conditions maintained at 20°C temperature, 90% humidity, 800 lux light intensity, and 700 ppm carbon dioxide concentration. The biological efficiency was calculated using the formula:

$$Biological\;efficiency=\frac{Fresh\;weight\;of\;fruiting\;body\;}{Dry\;weight\;of\;culture\;material}\times 100\%$$

The experiment was done under aseptic conditions and repeated three times using the same substrate ingredients, formula, and incubation rooms. The yield and biological efficiency reported are the average of the three trials.

### 2.4 Effect of different light quality on fruiting body formation

The PDA solid culture medium was divided into 250 mL Erlenmeyer flasks, each with a liquid volume of 100 mL, and sterilized under high pressure at 121°C for 30 min. A mycelium block was taken from the edge of the cultivated colony using a 9 mm hole punch, transferred to the center of the solid culture medium in the Erlenmeyer flask, and incubated in the dark at 25°C for 12 days.

Different light colors, including red, yellow, blue, green, purple, warm, and white light, were used for light cultivation at a temperature of 25°C and a light intensity of 800 lux. Each treatment was performed three times. The light quality was completely isolated with tin foil to prevent interference between different light qualities. The primordium and fruiting body differentiation of *P*. *abieticola* were observed and recorded under full light culture conditions.

### 2.5 Nutrient composition analysis in fruiting bodies

To determine the nutrient composition of the fruiting body, various analyses were performed. The amino acid content was determined using a Hitachi L-8900 Amino Acid Analyzer (Hitachi High-Tech Corporation, Tokyo, Japan) following the GB 5009.124–2016 protocol. Water-soluble total sugar was measured using a T6 new century UV-visible spectrophotometer (Xian Yima Optoelec Co., Ltd., Shaanxi, China), following the GB/T 15,672–2009 protocol. The fat content was determined using the Soxhlet extraction system of the Soxtec™ 2050 Automatic crude fat analyzer (FOSS Analytical AB, Höganäs, Sweden), following the GB 5009.6–2016 protocol. Protein was determined using the Kjeltec 8400 Kjeldahl nitrogen analyzer (FOSS, Hillerod, Denmark), following the GB 5009.5–2016 protocol. Trace elements were determined using the Agilent 7700X inductively coupled plasma mass spectrometer (Agilent Technologies Inc., Santa Clara, CA, United States of America), following the GB 5009.268–2017 protocol.

### 2.6 Mycelium incubation for transcriptome sequencing

For the mycelium incubation in transcriptome sequencing, 30 g of oak sawdust (broad-leaved trees) and larch sawdust (coniferous trees) were used as substrates with moisture content 60%. The substrates were then packed into three Petri dishes and sterilized at 121°C for 30 min. After cooling to room temperature, the spawn was inoculated into the Petri dishes and incubated in the dark at 25°C for 2 weeks. The mixture of mycelium and substrate was then used as the sequencing raw material.

### 2.7 RNA extraction and library construction

Following the manufacturer’s protocols, six samples were used to extract total RNA with TRIzol reagent (Life Technologies, NY, United States). The quality and integrity of the RNA were assessed with the Agilent 2100 Bioanalyzer (Agilent Technologies, Santa Clara, CA, United States). The RNA was fragmented, converted to cDNA, amplified, and purified with magnetic bead-based size selection. NEBNext UltraTM RNA Library Prep Kit for Illumina (NEB, Ipswich, MA, United States) was used to construct six cDNA libraries. The cDNA libraries’ quality and quantity were evaluated with the Agilent 2100 Bioanalyzer (Agilent Technologies, Santa Clara, CA, United States). Novogene Technologies (Tianjin, China) sequenced the libraries on an Illumina HiSeq 2500 platform (Illumina, San Diego, CA, United States). All the transcriptome sequences have been submitted to Figshare database (https://figshare.com/articles/dataset/Transcriptome_data/24454705).

### 2.8 Pretreatment of transcriptome sequencing data

Raw data from the transcriptome sequencing was filtered using Fastp software (Chen et al., 2018) to obtain clean data. The filtering process involved removing adaptor sequences, reads with unknown base content >10%, and low-quality reads. Mapping rates and other information were obtained using HISAT software (Kim et al., 2015) to compare clean reads with the reference genome.

### 2.9 Analysis of quantitative and differential expression genes

Several steps were followed to analyze the quantitative and differential expression of genes. First, Pearson correlation coefficients between pairs of samples were calculated using R software, and the results were visualized as heat maps. Next, Bowtie2 software (Langmead and Salzberg, 2012) was used to map clean reads to the reference genome sequence, and RSEM (Li and Dewey, 2011) was used to determine the gene expression level for each sample. DEGseq software (Wang et al., 2010) was then used to identify differentially expressed genes between the two samples using Poisson distribution analysis. Functional annotation for these genes was performed using Gene Ontology (GO) and Kyoto Encyclopedia of Genes and Genomes (KEGG) databases. Enrichment analysis based on the Phyper function was performed using R software, and a Qvalue <0.05 was considered a significant enrichment.

### 2.10 Verification of differentially expressed genes by qRT-PCR

To validate the results of RNA-Seq, the same RNA samples were used for qRT-PCR. The RNA was reverse transcribed into cDNA using the TransScript^®^ Green One-Step qRT-PCR SuperMix (Beijing, China). Each treatment was repeated three times. The Applied Biosystems™ StepOnePlus™ Real-Time PCR System (Applied Biosystems, Waltham, MA, United States) was used to collect data on the differentially expressed genes (DEGs). The 2-△△Ct method was used for relative gene expression analysis, with β-actinF GGA​GAA​GAT​TGG​CAT​CAC​ACA and β-actinR GAA​GAG​CGA​AAC​CCT​CGT​AGA serving as reference genes.

## 3 Results

### 3.1 Optimization of growth conditions for *P*. *abieticola* mycelium

#### 3.1.1 Optimization of carbon source

**FIGURE 1:**
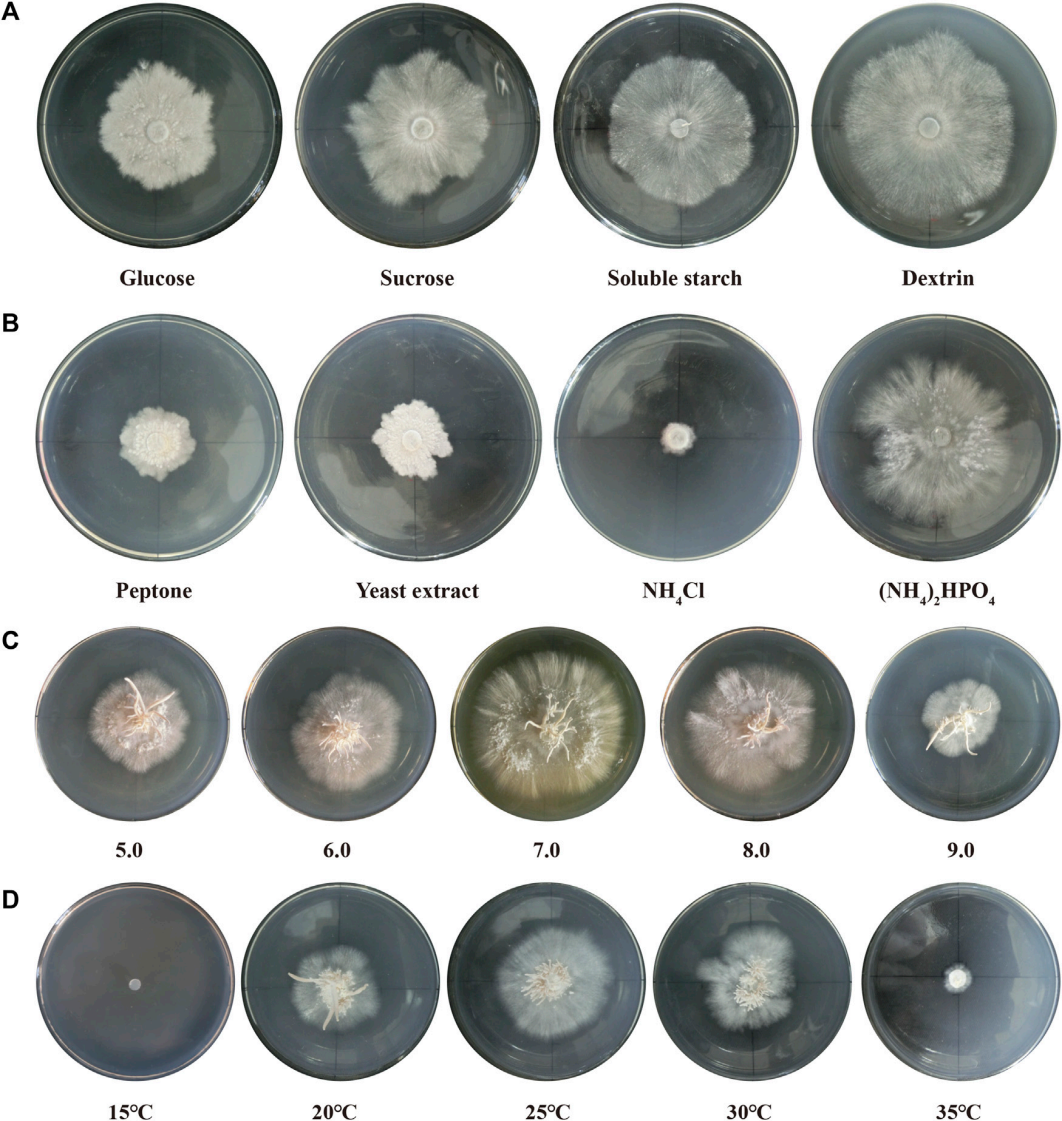
Optimization of mycelium culture conditions in *P*. *abieticola*. **(A)** Effect of different carbon sources on mycelial growth and activity. **(B)** Effect of different nitrogen sources on mycelial growth and activity. **(C)** Effect of different pH conditions on mycelial growth and activity. **(D)** Effect of different temperature conditions on mycelial growth and activity.

*P*. *abieticola* mycelia grew using four different carbon sources. Glucose resulted in slow growth and lower activity. When sucrose and soluble starch were used as carbon sources, the mycelia grew faster and had general activity. The growth rate of mycelia was slightly higher when using dextrin as the carbon source, resulting in dense mycelia and a regular edge of the colony. Therefore, dextrin was the most suitable carbon source, followed by soluble starch, sucrose, and glucose (Figure 1A; Supplementary Table S2).

#### 3.1.2 Optimization of nitrogen source

*P*. *abieticola* mycelia grew on the four nitrogen sources with glucose as carbon source. Diammonium hydrogen phosphate resulted in the fastest growth rate, twice as fast as peptone and yeast extract. The mycelium had good growth vitality and normal color, and its growth rate was significantly higher than other nitrogen sources. Therefore, (NH₄)₂HPO₄ was the most suitable nitrogen source, followed by yeast powder, peptone, and NH_4_Cl (Figure 1B; Supplementary Table S2).

#### 3.1.3 Optimization of pH

*P*. *abieticola* mycelia could grow under five different pH conditions. The mycelia grew the fastest at pH 7.0, with a significantly higher growth rate compared to other pH conditions. The edge of the colony was regular, and the mycelia vitality was high. The mycelia also showed good growth at pH 6.0 and pH 8.0. However, the mycelia grew slowly at pH 5.0 and 9.0, with irregular edges of the colony. Therefore, the most suitable pH value for the growth of *P*. *abieticola* mycelia was 7.0 (Figure 1C; Supplementary Table S2).

#### 3.1.4 Optimization of temperature

*P*. *abieticola* mycelia could not grow at 15°C. The mycelial growth rate was the fastest at 25°C, with a significantly higher growth rate than other temperature conditions. The edge of the colony was regular, and the mycelial vitality was high. The mycelia also grew well at 20°C and 30°C. However, the mycelia grew slowly at 35°C, with irregular colony edges. Therefore, the optimal temperature for mycelia growth of *P*. *abieticola* was 25°C, and the tolerance temperature range was determined to be 20°C–35°C (Figure 1D; Supplementary Table S2).

### 3.2 Effect of light quality on fruiting body development in *P*. *abieticola*

**FIGURE 2:**
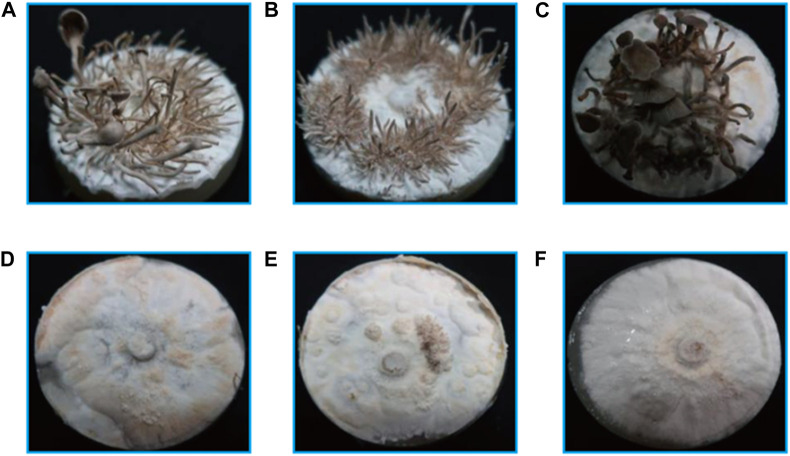
Fruiting body development under different light qualities in *P*. *abieticola*. **(A)** Red light; **(B)** Yellow light; **(C)** White light; **(D)** Violet light; **(E)** Green light; **(F)** Blue light.

Seven different light qualities (800 lux) were tested and significant effects were observed (Figure 2). The best growth and development state of the fruiting bodies were observed under white light, with more primordium and complete lamella and pileus formation. The mycelia of *P*. *abieticola* could differentiate into complete fruiting bodies in PDA solid medium under white light. Red light stimulated primordium formation, but the pileus was deformed, and no spores were observed. Yellow light promoted the formation of the maximum amount of primordium, but it couldn't directly induce the differentiation of the pileus. Green light stimulated the formation of the primordium and pigment. Purple and blue light conditions did not result in the formation of any primordium.

### 3.3 Domestication and cultivation of wild *P*. *abieticola* mushroom using different substrate formulas

**FIGURE 3:**
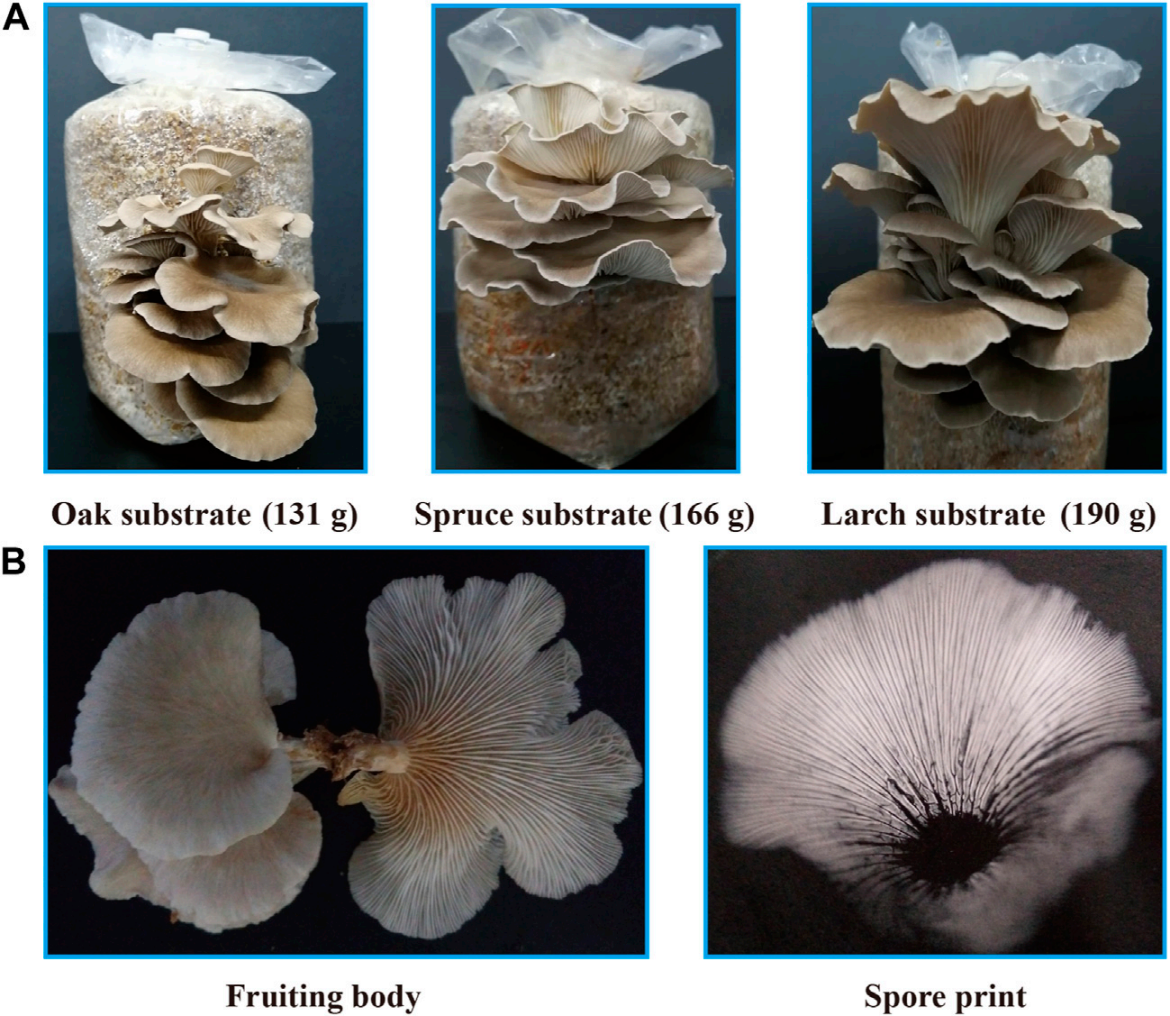
Fruiting body yield and phenotype of *P*. *abieticola* with different substrates. **(A)** Yield of the first flush with various substrates. **(B)** Phenotype of *P*. *abieticola* fruiting body with gray color, thin and brittle flesh, sparse gill spacing (mediotrastum), white spore print, and slightly yellow base.

The larch substrate had the highest average yield of the first flush (190 g) and biological efficiency (38.0%), while the average yield of oak and spruce substrates was 131 g (26.2%) and 166 g (33.2%), respectively (Figure 3A). Moreover, the oak substrate resulted in a thinner and lighter fruiting body phenotype, whereas the conifer substrates produced thicker and darker fruiting bodies (Figure 3B).

### 3.4 Nutrient composition of fruiting body in *P*. *abieticola*

Table 1 shows the nutrient composition of *P*. *abieticola* fruiting bodies, which are rich in protein (31.2%) and trace elements like calcium, iron, zinc, and selenium (Table 1; Supplementary Table S3). The fruiting bodies contain 23.93 g/100 g of 16 essential amino acids, with 7.93 g/100 g being essential. The essential to total and non-essential amino acids ratio was 0.33 and 0.50, respectively. They also contain 28.4% water-soluble sugars and a lower fat content than other edible mushrooms. The total water-soluble sugar and protein contents are higher than in other edible mushrooms (Zhang et al., 2017; Wang et al., 2018). The most abundant amino acids in the fruiting bodies are serine, glycine, valine, leucine, tyrosine, phenylalanine, lysine, histidine, and arginine.

**TABLE 1:** Comparisons of nutrient content on six edible fungi (g/100 g).

Nutritional components	*P. abieticola*	*P. ostreatus*	*P. placentodes*	*Agaricus*. *bisporus*	*L. edodes*	*F. velutipes*
Asp	2.49	1.33	2.63	1.97	1.43	0.78
Thr	1.28	0.83	1.81	1.10	0.95	0.66
Ser	1.43	0.81	0.99	1.10	0.85	2.45
Glu	5.31	3.49	3.13	4.25	5.47	2.11
Gly	1.18	0.81	0.94	1.01	0.76	1.18
Ala	1.71	1.58	1.26	2.27	0.74	1.24
Val	1.44	0.90	0.95	1.09	1.08	0.77
Met	0.05	0.27	0.16	0.40	0.49	0.12
Ile	0.73	0.59	0.81	0.81	0.72	0.54
leu	1.99	1.15	1.43	1.69	1.06	0.90
Tyr	0.72	0.46	0.59	0.58	0.23	0.83
Phe	1.05	0.45	0.79	0.51	0.60	0.71
Lys	1.36	0.77	0.85	1.08	1.06	1.09
His	0.57	0.37	0.40	0.50	0.42	0.35
Arg	1.58	0.73	1.06	1.12	0.91	0.58
Pro	0.99	0.88	0.87	2.22	0.57	0.64
Protein	31.2	22.3	30.1	31.1	30.1	24.5
Total sugar	28.4	21.2	-	12.2	14.4	16.6
Fat	1.3	5.6	2.7	2.5	3.6	2.5

Note: The value in each table represents the content per 100 g of dry product, “-” means the corresponding measurement result was not found.

### 3.5 Differential gene expression (DEG) analysis in *P*. *abieticola* mycelia under varied cultivation substrates

**FIGURE 4:**
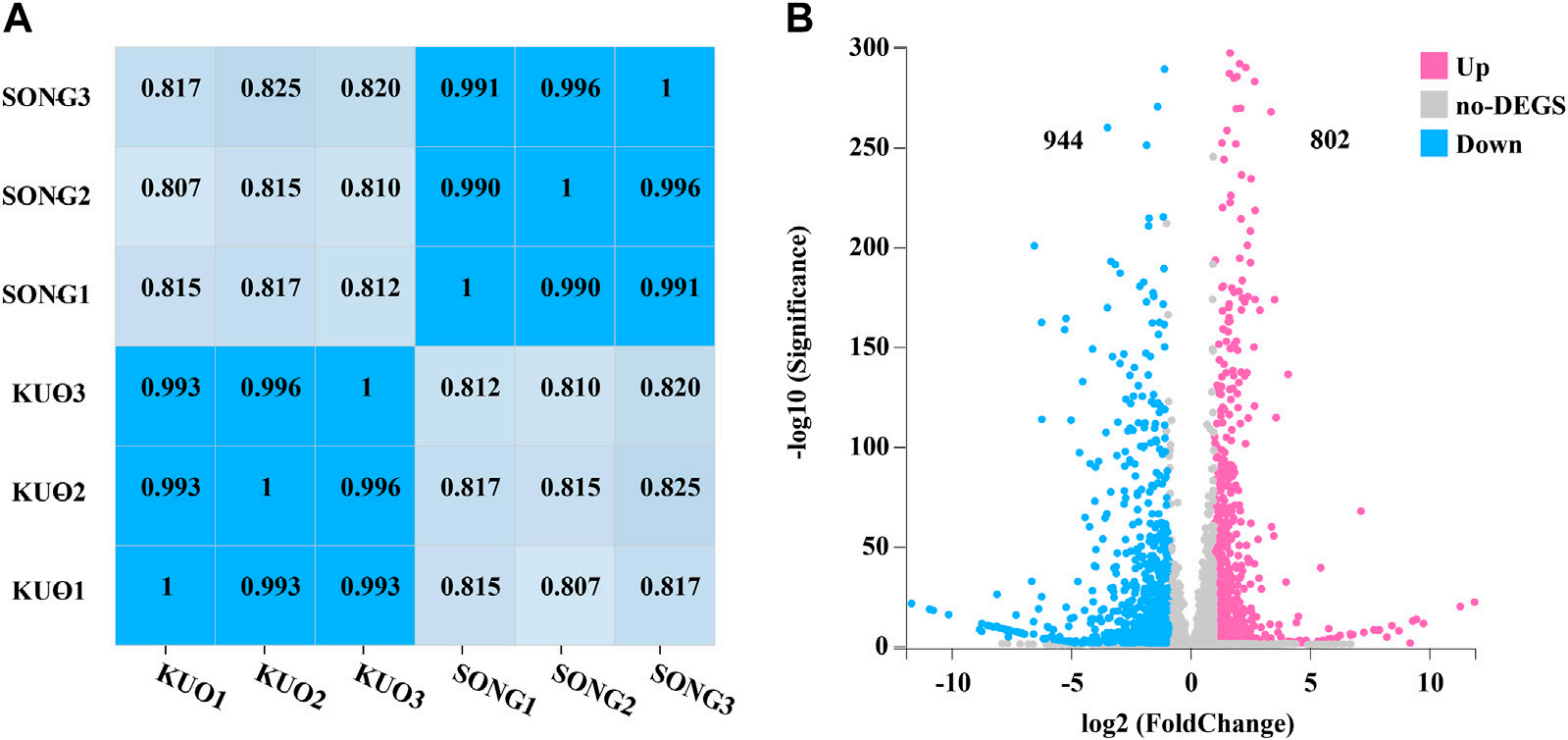
Differentially expressed genes analysis in mycelia of *P*. *abieticola* under different cultivation conditions. **(A)** The correlation analysis between different samples indicates the reliability and consistency of sequencing data and sampling. **(B)** The differential gene expressions (DEG) between mycelia in conifer and broadleaf tree substrates highlight the number of upregulated and downregulated genes and the significant differences between the two substrates.

DEGs in coniferous and broad-leaved substrate of *P. abieticola* mycelium were identified by transcriptome with the Hiseq 2500 platform. Each sample was repeated three times, resulting in a total of 11.81 G data. After filtering out low-quality reads and connectors, we obtained a high level of clean reads with Q30 > 95% and the three replicates of each substrate was over 99% (Figure 4A), indicating the reliability of subsequent analysis (Table 2). Then, the genome of *P. abieticola* genome downloaded from the MushDB database (Fu et al., 2022) was used as reference to identified DEGs with DEGseq software (*p* < 0.05). A total of 1746 differentially expressed genes were identified, accounting for 16.20%. Among them, 802 genes were upregulated, and 944 genes were downregulated in coniferous vs. broad-leaved trees (Figure 4B). Among the DEGs, 88 differentially significant genes with | log2 conifer vs. broadleaf | >5, of which 27 genes were upregulated in the coniferous substrate, and 61 genes were upregulated in the broad-leaved substrate. The above results indicate that the highly DEGs in the utilization of different substrates of *P. abieticola* were mainly related to the substrate of broad-leaved substrate.

**TABLE 2:** Sequence data and statistics of genome mapping results.

	Broadleaf1	Broadleaf2	Broadleaf3	Conifer1	Conifer2	Conifer3
Raw reads (M)	16.14	13.72	11.52	13.84	12.63	11.55
Clean reads (M)	16.07	13.66	11.46	13.75	12.55	11.49
Clean bases (Gb)	2.40	2.04	1.72	2.06	1.88	1.71
Q20 (%)	98.66	98.48	98.42	98.52	98.21	98.48
Q30 (%)	95.82	95.25	95.14	95.40	94.57	95.25
Clean reads Ratio (%)	99.55	99.56	99.48	99.40	99.39	99.51
Mapping (%)	96.13	96.17	95.89	96.36	95.72	95.81

### 3.6 Enrichment analysis of DEGs in *P*. *abieticola* mycelia grown in different substrates

#### 3.6.1 Enriched DEGs (1746)

**FIGURE 5:**
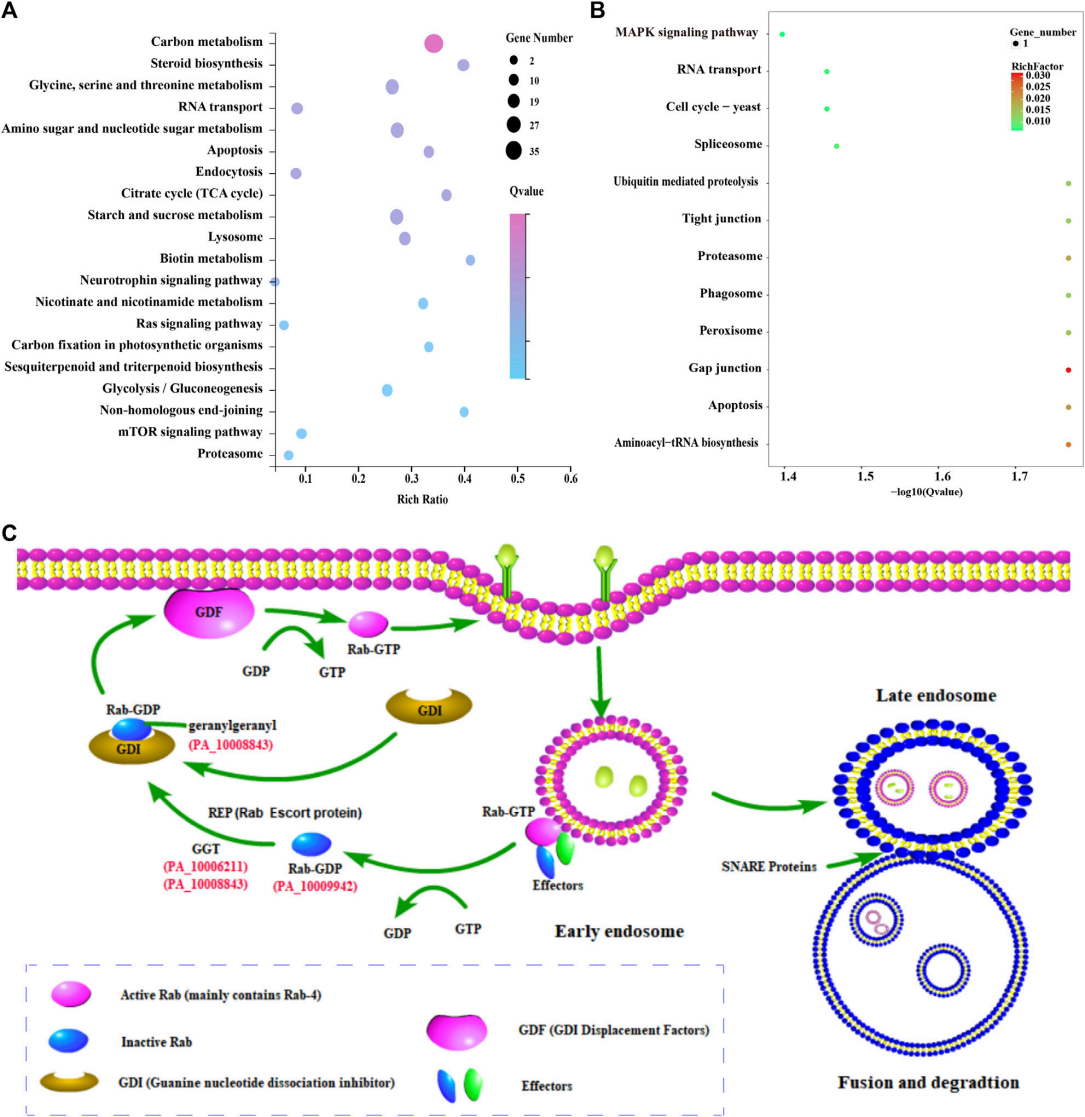
Enrichment analysis of differentially expressed genes and its possible endocytosis pathway in *P*. *abieticola*. **(A)** Top 20% enriched KEGG pathways of differentially expressed genes; **(B)** KEGG enrichment analysis of 27 highly expressed genes in conifer substrate; **(C)** Possible endocytosis pathway during *P*. *abieticola* substrate colonization, with upregulated key genes involved in the process with red color, such as Rab-4b, SNARE protein, and geranylgeranyl transferase.

The results showed that 265 and 540 functional genes were annotated in GO and KEGG databases, respectively. A total of 222 GO terms were enriched with a *p*-value <0.05, such as metabolic process, catalytic activity, oxidoreductase activity, hydrolase activity, acting on glycosyl bonds, and carbohydrate metabolic process. In addition, 43 metabolic pathways were enriched with a *p*-value <0.05, including carbon metabolism, starch and sucrose metabolism, Amino sugar and nuclear sugar metabolism, and Glycine, serine and threonine metabolism (Figure 5A). Sterol biosynthesis was also found to be significantly enriched, with squalene epoxidase, a key enzyme gene regulating sterol synthesis, highly expressed in the mycelia of conifer substrate.

#### 3.6.2 Enrichment of 27 upregulated expressed genes

Eight functional genes (the other 19 genes have no known function) and 82 GO terms were annotated in the GO database, including structural constituents of the cytoskeleton, microtubule, supramolecular complex, and acyl-CoA dehydrogenase activity. Similarly, the KEGG database contained ten metabolic pathway genes and 12 metabolic pathways, such as Gap junction, Aminoacyl tRNA biosynthesis, Proteasome, Spliceosome, Apoptosis, and MAPK signaling pathway (Figure 5B). Several metabolic pathways and upregulated expression genes related to stress resistance were significantly enriched.

#### 3.6.3 Enrichment of endocytosis-related genes

Certain key genes involved in endocytosis, such as Rab-4b, SNARE protein, and geranylgeranyl transferase, was found to be upregulated among the differentially expressed genes (Figure 5C). The genome of *P*. *abieticola* also contains other effectors related to this process. Notably, the differentially expressed endocytosis genes identified in this study were mainly related to the early endosome fast recycling pathway.

### 3.7 Differential expression of CAZYmes in *P*. *abieticola* mycelia cultivated in different substrates

**FIGURE 6:**
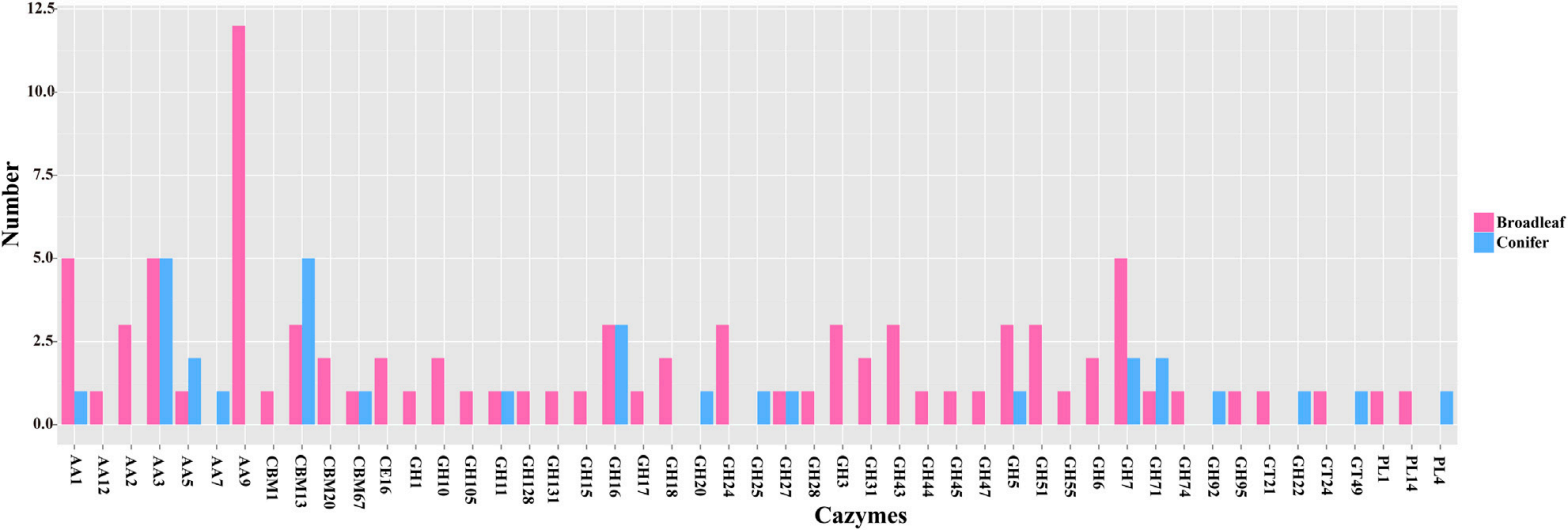
The number of differentially expressed CAZYmes genes in *P*. *abieticola* mycelia cultivated in the coniferous and broad-leaved substrate.

Our CAZYmes annotation of the *P*. *abieticola* genome revealed a total of 360 genes related to lignocellulose degradation, including 84 carbohydrate-binding modules (CBMs), 9 carbohydrate esterases (CEs), 182 glycoside hydrolases (GHs), 42 glycosyltransferases (GTs), 14 polysaccharide lyases (PLs), and 82 auxiliary activities (AAs). Out of 1746 differentially expressed genes, 119 were related to lignocellulose degradation, with 60 GHs accounting for 50.4% of them. The expression of CAZYmes genes varied significantly during the process of degrading the different types of lignocellulose, with GHs being the primary enzymes used for lignocellulose degradation in *P*. *abieticola*. Of the 119 DEGs, 42 and 19 different type CAZYmes were identified in broad-leaved and coniferous substrates, respectively. In this study, 87 differentially expressed CAZYmes were highly expressed in the broad-leaved substrate, and 32 CAZYmes were highly expressed in the coniferous substrate. Comparing the types of CAZYmes highly expressed in conifers and broad-leaved substrate, the genes only highly expressed in conifers were AA7, GH20, GH25, GH92, GT22, GT49, and PL4. AA9, AA3, and GH7 were the most annotated gene families in the differential expression CAZYmes gene family (Figure 6; Supplementary Table S4).

#### 3.7.1 Laccases and peroxidases

(MnP, LiP, VP, cytochrome c, etc.), primarily belonging to the AA1 and AA2 families of CAZymes, are known to be involved in the degradation of lignocellulose. Our analysis identified 6 differentially expressed genes from the AA1 family and 3 from the AA2 family in *P*. *abieticola* mycelia cultivated on different substrates (Supplementary Table S4). Among these, 5 laccase genes were highly expressed in the broad-leaved substrate, while 1 differentially expressed ferroxidase showed highly expression in coniferous substrates. Two manganese peroxidase and one cytochrome C peroxidase differentially expressed genes were highly expressed in broad-leaved trees (Supplementary Table S4).

### 3.8 Validation of gene expression patterns by qRT-PCR

**FIGURE 7:**
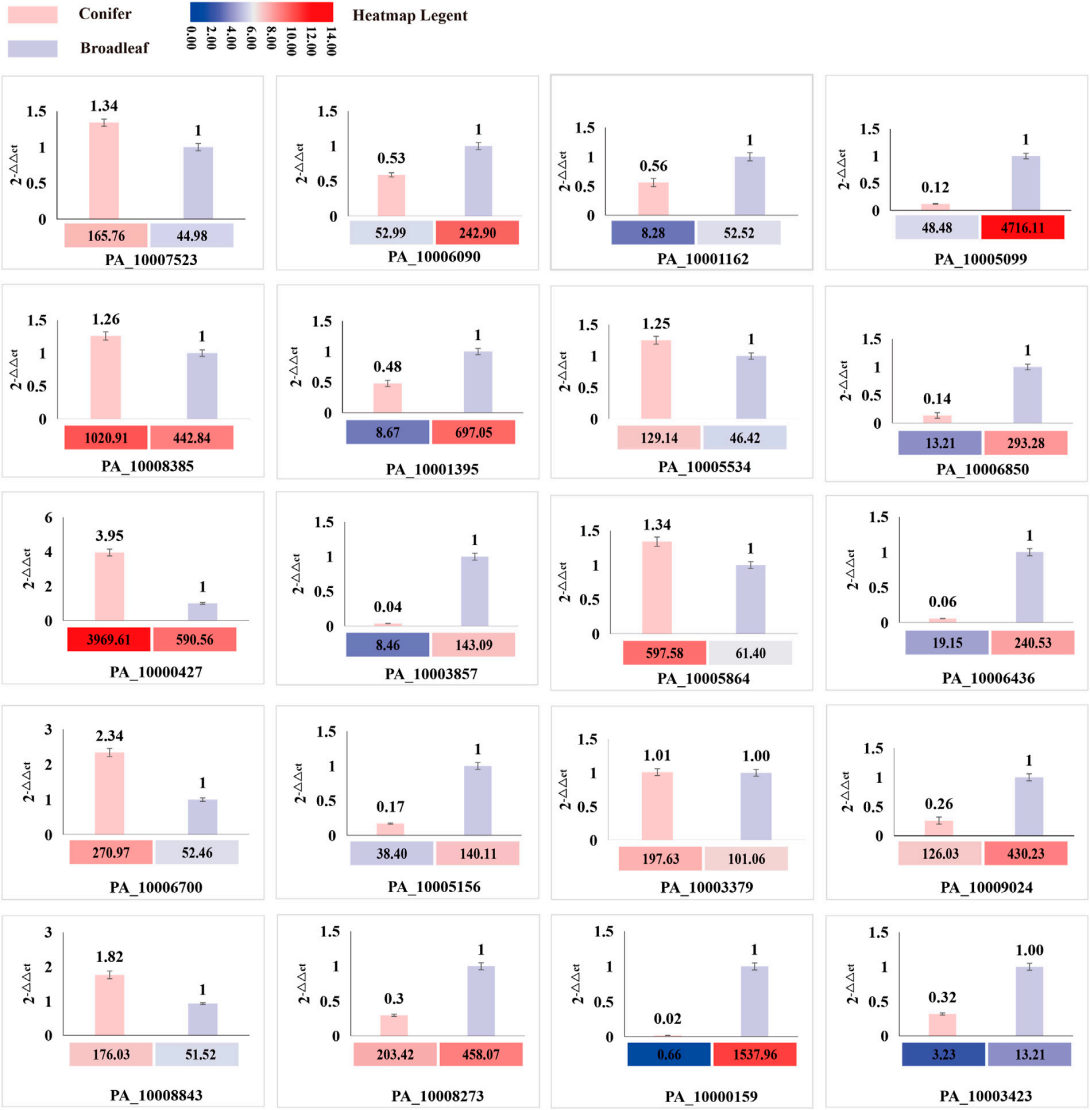
Comparison of the expression level of DEG genes between transcriptome and qRT-PCR. The histogram represents the expression level of qRT-PCR, and the heatmap represents the FPKM value of RNA sequencing analysis.

A total of 20 differentially expressed genes (DEGs) involved in CAZYmes, hydrophobin gene, transcription factor, cytochrome C reductase, manganese peroxidase, laccase, and squalene epoxidase were validated by qRT-PCR using β-actin gene as a reference and 2^-△△ Ct^ as the calculation method. The expression trend of these 20 genes in the coniferous and broad-leaved substrate was consistent with the transcriptome results, indicating the accuracy of transcriptome sequencing analysis results and their relevance to substrate utilization. These results are presented in Figure 7; Supplementary Table S5.

## 4 Discussion

The *Pleurotus* genus includes many edible species that are widely distributed and have significant nutritional and economic value (Petersen and Hughes, 1997; Dai et al., 2019; Li et al., 2020). While *Pleurotus ostreatus* and *Pleurotus pulmonarius* are the most commonly cultivated species, other wild *Pleurotus* species offer potential sources of functional foods, medicine, and biotechnological applications (Corrêa et al., 2016). In this study, we characterized the biological characteristics and nutrition profile of *P*. *abieticola* and established cultivation techniques for its domestication and commercialization. We also performed transcriptome analysis to understand the mechanisms for its utilization of conifers.

*P*. *abieticola* has a broad range of nutrient utilization capabilities and can grow well on both broadleaf and conifer substrates compared to other *Pleurotus* species (Deora et al., 2021; Muswati et al., 2021; Oyetayo et al., 2021). Due to its ability to grow on waste products from conifer trees, as well as agricultural and forestry residues that are abundant in China, *P*. *abieticola* shows great promise as a candidate for use in industries such as pharmaceuticals, textiles, biorefinery, bioremediation, cosmetics, and food packaging (Corrêa et al., 2016; Xiao et al., 2019). The yield and biological efficiency were the highest when *P*. *abieticola* was grown on larch sawdust substrate, which contains more lignin but less hemicellulose and cellulose. This is in line with the discovery that the biological efficiency of *P*. *ostreatus* was linked to the amount of lignin in the substrate, but inversely linked to the amounts of hemicellulose and carbohydrates present in the substrate (Koutrotsios et al., 2014). While *P*. *abieticola* may have a slower hyphae growth period than other *Pleurotus* species (Qi et al., 2000), its growth can be improved by adjusting its exposure to light. By controlling light exposure, it is possible to increase the production of the primordium. Additionally, cultivating *P*. *abieticola* under green light may be advantageous for the production of secondary metabolites. The study found that *P*. *abieticola* mycelia grow optimally when provided with dextrin powder as a carbon source and (NH_4_)_2_HPO_4_ as a nitrogen source, with a pH of 7.0 and a temperature of 25 °C. These optimal conditions are within the range for most *Pleurotus* species (Corrêa et al., 2016; Mahari et al., 2020). The mushroom’s preference for dextrin as a carbon source may be related to the presence of genes encoding dextrinase and alpha-amylase enzymes, while (NH_4_)_2_HPO_4_ as a nitrogen source may stimulate the production of enzymes involved in nutrient uptake and metabolism, as well as secondary metabolites like alkaloids or pigments (Ribeiro et al., 2008).

Understanding the biological characteristics of mushrooms is crucial for their commercial cultivation. It can aid in selecting appropriate substrates, optimizing growth conditions, and improving yields while reducing operational costs. Furthermore, knowledge of mushroom biology can provide valuable insights into their ecology and interactions with other organisms in their environment. The biological characteristics of *P*. *abieticola* offer insight into its adaptability and growth preferences, which can facilitate high-quality production and resource utilization.

To optimize the commercial cultivation of *P*. *abieticola*, we suggest several recommendations. These include using controlled packaging sizes for the spawn to promote consistency in growth, avoiding light during mycelium development, and adopting a clinker cultivation mode to minimize the risk of contamination. Additionally, scratching the spawning surface and removing aged spawn skin during mushroom production can improve primordium formation, the initial stage of mushroom growth. Implementing these recommendations may help increase the yield and quality of *P*. *abieticola* mushrooms in commercial settings.

The nutritional composition of *P*. *abieticola* suggests that it has the potential to be a valuable addition to the food industry. This is because it contains high amounts of protein, important amino acids, total sugars, and small amounts of essential trace elements like calcium, zinc, and selenium. It may be a good alternative for vegetarians and those seeking protein-rich food sources. Further research may be needed to fully understand the nutritional benefits of *P*. *abieticola* and explore its potential use in developing new food products.

Transcriptome analysis is a powerful tool used extensively in biological research to explore the molecular level of organism growth, development, stress response, and functional gene mining (Fu et al., 2017; Ye et al., 2021; Yang et al., 2022a; Wang et al., 2022). In this study, transcriptome sequencing of *P*. *abieticola* mycelia on different substrates revealed differentially expressed metabolic pathways and enzyme genes related to stress resistance, including the crucial cell membrane stability and endocytosis pathways. Squalene epoxidase, a gene encoding a key enzyme involved in the biosynthesis of cell membrane sterols, was highly expressed in conifer substrates (Nakano et al., 2007). Inhibiting this enzyme has been a key strategy in developing antifungal drugs (Wu et al., 2013; Sagatova, 2021), and essential oils have been shown to inhibit sterol synthesis in *Candida* (Eugénia et al., 2017). These results suggest that the ability to tolerate the effects of conifer components on cell membranes may be a crucial factor for the successful cultivation of *P*. *abieticola* on conifer substrates. However, the gene expression multiples detected by qRT-PCR was similar, which need further in-depth research in the future.

Fungal endocytosis is critical in facilitating nutrient uptake, establishing symbiotic relationships with host plants, and detecting and responding to environmental signals (Rollenhagen et al., 2021; Zheng et al., 2021; Chen et al., 2022a). In addition, it provides an alternative way for the mushroom to acquire nutrients from conifer substrates without damaging the mycelia due to the presence of tannins, resins, and other bioactive compounds found in conifers (Wu et al., 2021; Chen et al., 2022b). Therefore, endocytosis is an essential process for the successful colonization of conifers by *P*. *abieticola*. The significantly upregulated expression of key genes such as *Rab*-*4* and geranylgeranyl transferase in the endocytosis process provides new insights into the genetic basis of *P*. *abieticola’s* resistance to conifer substrates. The study revealed significant enrichment in phagocytosis, indicating that *P*. *abieticola* has an enhanced ability to remove damaged cells. In addition, the aminoacyl tRNA synthetase and MAPK signal transduction pathways were identified, which have been associated with stress resistance in various organisms (Sun et al., 2014; Mohler and Michael, 2017). However, the growth and development of organisms are intricate processes, and further systematic research is necessary to fully understand the adaptive mechanisms of *P. abieticola* to coniferous substrates.

The results of this study provide further evidence of the crucial role of CAZYmes in the utilization of lignocellulose as a substrate for *P*. *abieticola* (Cui et al., 2019). The identification of 119 differentially expressed CAZYmes genes related to lignocellulose degradation emphasizes the importance of these enzymes in the degradation of conifer substrates. Specifically, the study identified 13 GHs among the 32 highly expressed CAZYmes genes in the conifer substrate, including 3 GH16 and 2 GH7 genes, with the other GH types represented by only 1 gene each. These findings are consistent with previous studies that have identified β-glucosidase and β-glucanase as key enzymes for the early decomposition of conifer substrate (MacDonald et al., 2011; Šnajdr et al., 2011). The study revealed the presence of highly expressed AA9 genes responsible for cellulose degradation in the broad-leaved tree substrate. This finding aligns with MacDonald’s study results, which used *Phanerochaete carnosa* as the subject material (MacDonald et al., 2011). These findings suggest that *P*. *abieticola* may have different enzymatic requirements for different types of lignocellulosic substrates. Further research is necessary to delve into the underlying mechanisms of this differential enzyme expression and its implications for the cultivation of *P*. *abieticola* on different substrates.

Laccase and peroxidase are key enzymes in the degradation of lignin within the CAZYmes family. Extensive research has been conducted to investigate their ability to break down lignin through chemical synthesis and fungal secretion (Avanthi and Rintu, 2016; Jha, 2019). The current study identified that five peroxidase genes and five laccase genes were differentially expressed. It is interesting to note that among the peroxidase and laccase genes analyzed, four peroxidase genes showed high expression in the broad-leaved substrate, while all five laccase genes showed high expression in the same substrate. This finding is in line with the high lignin content of broad-leaved trees. However, a separate study by Żółciak (2019) found low activity levels of laccase, lignin peroxidase (LiP), and manganese peroxidase (MnP) in sapwood and new wood (Żółciak, 2019). Additionally, the study found that the multifunctional peroxidase (VP) expression activity was high, but the degradation ability to guaiacyl was weak when studying the degradation ability of peroxidase (LiP, MnP, VP) and laccase (Laccase) in *P. abieticola* to sapwood and heartwood of Norway spruce. Since the type of lignin in conifers is predominantly guaiacyl lignin (Longe et al., 2018; Yang et al., 2022b), the weak ability of *P. abieticola* to degrade guaiacyl and the absence of differential expression of VP in this study suggest that VP is not the primary enzyme for coniferous degradation. As a result, it is hypothesized that laccase and peroxidase are not the primary enzymes adapted to the coniferous substrate of *P*. *abieticola*.

These findings have significant implications for our understanding of the mechanisms involved in lignin degradation, especially in the context of biomass utilization and bioenergy production. They suggest that other enzymes or mechanisms may be involved in lignin degradation in conifers. Further research is needed to explore these potential mechanisms and optimize lignin degradation for various applications. Additionally, these findings significantly impact our comprehension of the diverse microbial communities involved in lignin degradation in different environments and how they adapt to different types of lignin and substrates.

## 5 Conclusion

In this study, we have successfully characterized the biological features, domestication potential, and nutritional value of *P*. *abieticola* as a promising edible mushroom with great commercial value. Our transcriptome analysis of coniferous substrate tolerance has provided insights into the metabolic pathways related to immunity and protein translation that may contribute to the fungus’ ability to adapt to coniferous substrates, a challenging substrate. Moreover, our identification of endocytosis metabolism as potential key factors in conifer adaptation may facilitate the valorization of this wild mushroom in biotechnological applications. Additionally, our findings on the differential expression pattern of CAZYmes have shed light on the molecular mechanisms underlying substrate degradation in *P*. *abieticola*, thereby expanding the pool of cultivated mushrooms and enhancing their potential contribution to the development of the food and biotechnology industries. Future studies may focus on exploring the secondary metabolite production of *P*. *abieticola*, which could further augment its economic value and biotechnological applications.

## Data Availability

The datasets presented in this study can be found in online repositories. The names of the repository/repositories and accession number(s) can be found in the article/Supplementary Material.
